# Semantic enhanced for out-of-distribution detection

**DOI:** 10.3389/fnbot.2022.1018383

**Published:** 2022-11-03

**Authors:** Weijie Jiang, Yuanlong Yu

**Affiliations:** College of Computer and Data Science, Fuzhou University, Fuzhou, China

**Keywords:** out-of-distribution detection, semantic enhancement, label smoothing, multi-perspective, deep learning

## Abstract

While improving the performance on the out-of-distribution (OOD) benchmark dataset, the existing approach misses a portion of the valid discriminative information such that it reduces the performance on the same manifold OOD (SMOOD) data. The key to addressing this problem is to prompt the model to learn effective and comprehensive in-distribution (ID) semantic features. In this paper, two strategies are proposed to improve the generalization ability of the model to OOD data. Firstly, the original samples are replaced by features extracted from multiple “semantic perspectives” to obtain a comprehensive semantics of the samples; Second, the mean and variance of the batch samples are perturbed in the inference stage to improve the sensitivity of the model to the OOD data. The method we propose does not employ OOD samples, uses no pre-trained models in training, and does not require pre-processing of samples during inference. Experimental results show that our method enhances the semantic representation of ID data, surpasses state-of-the-art detection results on the OOD benchmark dataset, and significantly improves the performance of the model in detecting the SMOOD data.

## 1. Introduction

Deep neural networks have been very successful in identifying images, but when faced with out-of-distribution (OOD) data, they will be identified as in-distribution (ID) data with high confidence (Hendrycks and Gimpel, 2017). This is limiting the application of deep neural networks in high safety and reliability areas such as medical diagnosis (Fernando et al., 2021) and autonomous driving (Wang et al., 2015). To better study and improve the generalization performance of deep neural networks on OOD data, Hendrycks and Gimpel (2017) formalized the OOD detection task. The task demands that the model is able to first distinguish whether the input data is ID data or not during the inference process, and then classify the ID data accurately.

The mainstream research suggests that the performance of OOD detection depends on the features extracted by the model. In addition to using the conventional cross-entropy loss to optimize the extracted features (Hendrycks and Gimpel, 2017; Lee et al., 2018; Liang et al., 2018; Hsu et al., 2020; Sastry and Oore, 2020; Lin et al., 2021), another part of the work hopes to improve the OOD generalization performance of the features, such as constraining the distribution of the data in the feature space (Dhamija et al., 2018; Techapanurak and Okatani, 2019; Hassen and Chan, 2020; Zaeemzadeh et al., 2021), extra use of OOD data (Hendrycks et al., 2019a; Papadopoulos et al., 2021), and the use of and multi-task learning (Hendrycks et al., 2019b; Perera et al., 2020; Winkens et al., 2020).

The existing state-of-the-art method (Hsu et al., 2020; Sastry and Oore, 2020) shows excellent performance on the OOD benchmark dataset, but still shows poor performance on the same manifold OOD (SMOOD) data (Liang et al., 2018), i.e., with a small maximum mean distance (MMD) distance from the ID data. The essential reason is that the model contains only limited semantic information in the extracted features, the supervised learning-based feature extraction approach focuses only on those features that minimize the loss during the learning process (Winkens et al., 2020). In this process, the semantic information contained in the extracted features is only “partial” and “one-sided” with respect to the original sample. On the other hand, the model learns using both foreground and background information of the sample, so the extracted features also contain both semantic and non-semantic information of the sample. For example, non-semantic information such as sample mean and variance are easily found in the SMOOD data. When the model encounters the above SMOOD data without learning enough effective semantic discrimination information, it is easy to misclassify the OOD data as ID data.

The key to solving the above problem is that the model extracts more essential semantics to distinguish features from similar semantics during the learning process. In the popular supervised learning framework, the features learned by the model depend on the supervised information given with the prior. For the same data, the model learns under different supervised information and it will extract features with differences. If these various supervised information are representations of the same semantics, then the model will capture representations of the same semantics under different supervised “perspectives.” For example, in a binary classification problem, the label description of the positive sample can be [1, 0] or [0.9, 0.1]. Usually, both labels can yield satisfactory models, indicating the representation of the positive sample semantics under different supervised information “perspectives,” respectively. For ease of reference, the term “semantic perspective” is used later to denote a supervisory information representation of semantics.

**Figure 1:**
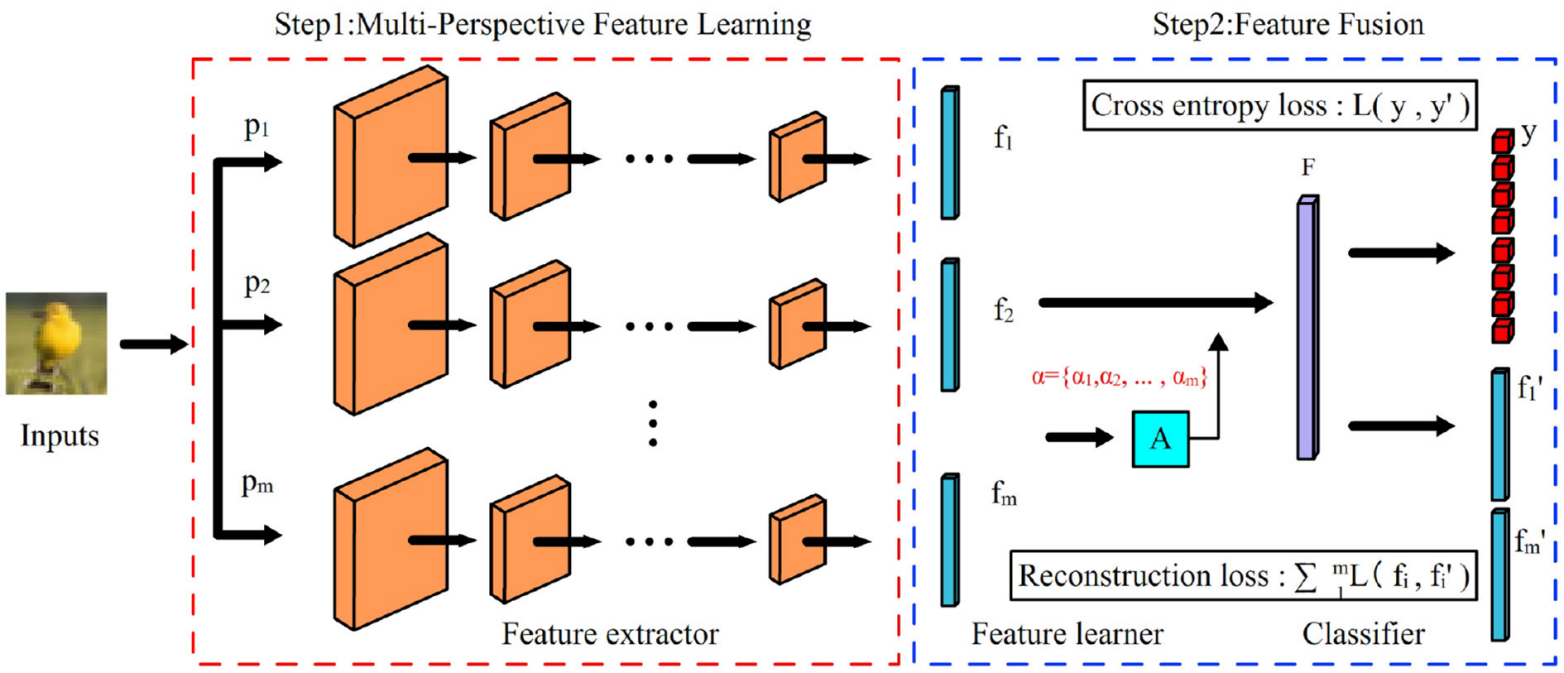
Semantic enhanced OOD detection model. The model consists of three parts: a feature extractor, a feature learner, and a classifier. The feature extractor extracts the features *f* = {*f*_1_, *f*_2_, ..., *f*_*m*_} of the input samples with the given *m* “semantic perspectives” *p* = {*p*_1_, *p*_2_, ..., *p*_*m*_}, and the feature learner fuses the *m* features in *f* to obtain the richer and more comprehensive features *F*, and the classifier obtains prediction results based on the features *F*.

Inspired by the above “semantic perspective,” we found that we can learn the same data from several different “perspectives” to obtain a more “comprehensive” semantic feature. Based on this motivation, we propose a semantic enhancement approach for OOD data detection, using features from multiple “semantic perspectives” to enhance the representation of semantic information in samples. The overall framework is shown in Figure 1. The model consists of three parts: feature extractors, multi-perspectives feature learner, and classifier. The training process of the model is divided into two stages. In the first stage, the feature extractor is trained, and the model is optimized for each given “semantic perspective,” and the optimized feature extractor is able to learn the features of each corresponding “semantic perspective.” In the second stage, the parameters of the feature extractor are not changed, the multi-view feature learner and classifier are trained. In this stage, instead of using the original samples, the “multi-perspective” features obtained by the feature extractor are directly used instead of the original samples.

In addition to semantic enhancement through multiple “semantic perspectives,” we also propose the self perturbing batch normalization (SPBN) method for open set scenarios. Perturbation is a common technique (Lee et al., 2018; Liang et al., 2018; Hsu et al., 2020) in OOD testing and is usually applied only in the inference phase of the model. Specifically, the test sample *x*_*i*_ needs to be preprocessed before inference, and the preprocessed $\tilde{x_{i}}=x_{i}-\varepsilon \text{ s i g n}\left[\nabla_{x_{i}}\log\left({ŷ}_{i,h}\right)\right]$, where ŷ_*i, h*_ is the predicted output of sample *x*_*i*_ with respect to the *h*th class and ∇_*x*_*i*__log(ŷ_*i, h*_) is the gradient of log(ŷ_*i, h*_) with respect to *x*_*i*_, ε is a hyperparameter that represents the magnitude of the perturbation. Input perturbation of test samples in the inference phase of the model can change the output of the model, and the two types of test data, ID and OOD, do not change to the same extent after perturbation. Using this different level of change can improve the model's ability to detect OOD data. In addition to preprocessing the input samples to change the output, the output can also be changed by altering the parameters in the propagation process. The parameters of the batch normalization (BN) layer (Ioffe and Szegedy, 2015) record statistical information about the distribution of ID data that is naturally different from the OOD data distribution. Inspired by this, we propose the SPBN method to influence the output by changing the parameters (i.e., mean and variance) of the BN layer. Specifically, while standard BN uses fixed mean and variance during inference, SPBN uses the mean and variance of the current batch sample. In contrast to the previous approach where a one-time perturbation was performed at the input, our approach perturbs at all layers of the network. In our approach, the statistical values of the ID test batch data differ less from the fixed statistical values used in the standard BN and are subject to less perturbation than the OOD data. SPBN is applied only in the feature learner, which on the one hand still maintains accurate prediction of the ID data; on the other hand, it increases the difference in prediction results between the ID and OOD data.

We validate the proposed method on OOD datasets of different scales and types, respectively, and the experimental results show that our method not only obtains more comprehensive semantic features, but also effectively enhances the ability of the model to detect OOD and SMOOD data. The contributions of this paper are mainly in two aspects:

- A semantic enhancement method based on multiple “semantic perspectives” is proposed to effectively improve the generalization performance of the model on OOD data. Compared with existing methods, the proposed approach is able to learn more “comprehensive” semantic features, which not only improves the accuracy of ID data, but also enhances the detection of OOD and SMOOD data, and exceeds the state-of-the-art performance on OOD benchmark datasets.
- We propose SPBN method for open set scenarios, which can be applied to OOD detection to maintain the semantics of the data better while improving the efficiency of detecting OOD data. SPBN method exploits the statistical differences between ID and OOD data to improve the performance of the model and does not require prior acquisition of the gradient of the test data or careful selection of the hyperparameters of the perturbation magnitude in the process of use.

## 2. Related work

Hendrycks proposed an Area Under the Receiver Operating Characteristic curve (AUROC) based performance metric and benchmark dataset for the OOD detection task, and using the maximum discriminant ID of the prediction probability of deep neural networks (Hendrycks and Gimpel, 2017). Thereafter, Liang et al. (2018) proposed a higher standard evaluation metric True Positive Rate of 95% (TPR95) and proposed the ODIN (Out-of-DIstribution detector for Neural networks) method. ODIN uses two strategies, temperature scaling and sample perturbation, to enhance the detection performance of the model for OOD samples during the inference process. Temperature scaling reduces the effect of the softmax function on sample overconfidence, and sample perturbation can further increase the difference between the prediction results of ID samples and OOD samples. However, ODIN requires OOD data as the validation set when calculating the magnitude hyperparameters of the perturbation. Hsu et al. (2020) reduced this requirement in the method GODIN (Generalized ODIN) by using only the ID dataset in the search for the perturbation magnitude, i.e., the same perturbation magnitude is used for all detection data (both ID and OOD data). GODIN also automatically learns the parameters of temperature scaling, which greatly improves the performance of the model on the benchmark dataset.

Another group of approaches motivates the network to learn more robust features from a feature learning perspective. Dhamija et al. (2018) starts from the motivation of separating ID and OOD data by concentrating the OOD data near the origin of the feature space and constraining the ID data away from the central origin to maintain class spacing. Hassen and Chan (2020) proposed two loss (intra spread—inter separation loss) based on Fisher Criteria wishing to increase class spacing and reduce intra-class spacing, by modifying the objective function to achieve the constraint of features; Techapanurak and Okatani (2019) used cos similarity instead of inner product as the basis of data categories, and reduced the influence of softmax function by compressing the range of logits. Zaeemzadeh et al. (2021) discriminates OOD data by mapping ID data to a union of one-dimensional space. To allow features to express richer information about ID data, Hendrycks et al. (2019a) and Perera et al. (2020) combine self-supervised learning into the training process of the model.

In addition to using the maximum of the predicted probabilities, some methods use proprietary OOD detectors to distinguish OOD samples. The detectors are usually trained to obtain them based on the features extracted by the classifier. Lee et al. (2018) uses Gaussian discriminant analysis to model the class of features in each layer of the network to obtain the martingale distance for each test sample, and uses the martingale distance to detect OOD data. Similarly, Winkens et al. (2020) uses the gram metric instead of the Mahalanobis distance and shows excellent performance in the detection of far OOD data types. Lin et al. (2021) compute and detect OOD data by selecting some of the network layer features based on OOD data types. DeVries and Taylor (2018) uses a detector to learn the confidence of the classifier prediction results, the detector shares features with the classifier and trains and learns simultaneously, and finally detects the OOD samples based on the results of the confidence; similar to the this, Corbière et al. (2019) uses an independent network to directly predict the maximum probability value of the test samples. Another line of research assumes that auxiliary data sets are available during the training process. For example, Hendrycks et al. (2019a) let classifiers learn OOD example samples before generalizing to other types of OOD data; Mohseni et al. (2020) uses auxiliary data to train OOD detectors; and more recently, Fort et al. (2021) and Koner et al. (2021) fine-tune and improve on the pre-trained Transtormer model.

## 3. Approach

### 3.1. Problem statement

Given the training set $D^{tr}={\left\{\left(x_{i}^{tr},y_{i}^{tr}\right)\right\}}_{i=1}^{N}$ and test set *X*^*te*^, where $X^{tr}={\left\{x_{i}^{tr}\right\}}_{i=1}^{N}$ is sampled from the distribution *P*_*in*_, $y_{i}^{tr}\in \left\{1,2,\ldots ,C\right\}$ is the label from C categories corresponding to $x_{i}^{tr}$, *X*^*te*^ is sampled from a mixture of *P*_*in*_ and *P*_*out*_ distributions, *P*_*out*_≠*P*_*in*_. The goal of OOD detection is to use *D*^*tr*^ to obtain a model *f* and *f* to determine whether $x_{j}^{te}\in X^{te}$ comes from *P*_*in*_ or *P*_*out*_, and for $x_{j}^{te}\in P_{in}$, the label ŷ_*j*_ of $x_{j}^{te}$ will be further predicted.

### 3.2. Training

The method in this paper is shown in Figure 1. The training process is divided into two stages: the first stage trains the feature extractor, which learns features from several different “semantic perspectives,” and the parameters of the feature extractor are not changed after training. The second stage trains the multi-perspective feature learner and classifier, in which the original samples are not used, but the “multi-perspective” features obtained by the feature extractor are directly used instead of the original samples. Section 3.2.1 provides details on how to obtain supervised information under different “semantic perspectives” by using label smoothing regularization, and the corresponding models; Section 3.2.2 presents the fusion network with multiple features and the training process.

#### 3.2.1. Feature extraction

In this stage, the model is required to learn features from several different “semantic perspectives,” and the greater the difference in “semantic perspectives,” the richer the semantics can be learned. In supervised learning, labels are usually used to express the semantic information of samples, and different expressions of labels in the objective function represent different “semantic perspectives.” Since samples from different classes tend to have similar visual elements to each other, we introduce the between-class smoothing assumption, assuming that the samples are in high-density regions of the true class and also in low-density regions of other classes with uniform probability. According to the assumption, the smoothed one-hot labels can be used as the representation of the labels under different “semantic perspectives.” Different smoothing prior parameters represent different “semantic perspectives,” and the smoothing coefficients are sampled uniformly from [0, 0.5] to ensure the validity and correctness of the “perspectives.” By this way, on the one hand, these smoothing coefficients do not exceed 0.5, so that the probability of the correct class is greater than the sum of the probabilities of all other classes, ensuring the correctness of the “perspective”; On the other hand, the use of uniform sampling allows the smoothing coefficients to be spaced as far apart as possible, increasing the difference in “perspective.”

Smoothing of labels is a model regularization technique that can effectively prevent model overfitting (Szegedy et al., 2016) and calibrate the confidence of the model predictions (Müller et al., 2019). Label smoothing requires specifying the smoothing factor ϵ and the prior distribution *u*(*h*) of the labels in advance. The prior *u*(*h*) generally uses a uniform distribution in practice, and the smoothed labels ${\left(y_{i}^{tr}\right)}^{\prime }$ are as follows (1),


(1) $$\begin{array}{l} {\left(y_{i}^{tr}\right)}^{\prime }=\left(1-\epsilon \right)\delta_{h,y_{i}^{tr}}+\epsilon /C \end{array}$$

where $\delta_{h,y_{i}^{tr}}$ is the Dirac delta, which equals 1 for $h=y_{i}^{tr}$ and 0 for $h\neq y_{i}^{tr}$. The probability of the *hth* class of the smoothed labels is no longer 1, and the probabilities of the remaining classes are not 0. With smoothed labels, the model does not need to overlearn semantically irrelevant features in the process of fitting the labels in order to obtain limit values like 1 or 0. Thus, the smoothed labels can avoid model overfitting. However, unsuitable smoothing coefficients may also lead to poorer performance of the model. Getting the appropriate smoothing coefficients is still an open research problem.

Cross entropy is a metric used to measure the difference in distribution. Larger values represent larger differences in distribution and vice versa. In neural networks, cross entropy is often used to measure the discrepancy between predicted and labeled true values, and this discrepancy is used as a loss function to guide the training of the network. Moreover, using the cross-entropy loss minimization objective function, small batch gradient descent can be used to optimize the parameters and improve the convergence speed. For the smoothed label $y_{i}^{\prime }=\left(y_{i,1},y_{i,2},\ldots ,y_{i,C}\right)$ and the output prediction ŷ_*i*_ = (ŷ_*i*, 1_, ŷ_*i*, 2_, ..., ŷ_*i, C*_) the cross-entropy loss is given by (2):


(2) $$\begin{array}{l} L_{ce}\left(y_{i}^{\prime },{ŷ}_{i}\right)=-\overset{C}{\underset{h=1}{\sum }}y_{i,h}^{\prime }\log\left({ŷ}_{i,h}\right). \end{array}$$

#### 3.2.2. Feature learning and classification

The feature learning stage uses a multilayer perceptron to fuse features *f* = {*f*_1_, *f*_2_, ..., *f*_*m*_} under the “perspective” to produce a global feature *F* with “comprehensive” semantic information, and finally use *F* for classification. In measuring the similarity between features F and class *h*, our approach discards the conventional inner product and uses a more compact cosin similarity, the calculation of logits *z*_*h*_ as shown in Equation (3):


(3) $$\begin{array}{l} z_{h}=\frac{w_{h}^{T}F}{\left||w_{h}^{T}||\cdot ||F|\right|} \end{array}$$

where *w*_*h*_ is the weight corresponding to the class *h*. Compared with the conventional use of inner product as logits, Equation (3) normalizes the logits. The normalized logits are constrained to values between (0, 1), preventing the network from rapidly increasing the weights to reduce losses and falling into local optimal points. Techapanurak and Okatani (2019) and Hsu et al. (2020) shown that this regularization-like mechanism can avoid the overfitting of the model to ID data and enhance the generalization of the model to OOD data.

In order to make the feature learner pay more attention to the features under good “perspectives,” we add an attention mechanism to feature learning, i.e., first mapping the features *f* linearly to an *m*-dimensional vector α, then using the softmax function to normalize α to obtain the attention weights under each “perspective,” and using the weighted features to calculate *F*. Finally, a linear reconstruction regular term is added to the feature learner in our method, requiring *F* to be able to linearly reconstruct *f*, which is used to balance the undesirable effects of the nonlinear transformation. The loss corresponding to the reconstruction regular term is calculated using the *l*_2_-loss.

Thus, the total loss of the feature learner is expressed as (4):


(4) $$\begin{array}{l} L=L_{ce}\left(y,y^{\prime }\right)+\lambda L_{rec}\left(f,f^{\prime }\right) \end{array}$$

where *y* is the one-hot label of the sample, *y*′ is the prediction result of the sample, *f* is the feature under different “perspectives,” *f*′ is the reconstructed feature. *L*_*ce*_ is the cross-entropy loss function, Calculated with Equation (2). *L*_*rec*_ is the *l*_2_ loss function, $L_{rec}=\underset{i}{\sum }{\left(f_{i}-f_{i}^{\prime }\right)}^{2}$. λ is the balance factor to balance the importance of the two objectives. Increasing the weight of λ means that more information is needed to maintain the original feature f. However, this may destroy the model's learning of the ID data, and a smaller λ is chosen in our approach.

### 3.3. Inference

The model combines SPBN and temperature scaling in the inference phase to obtain the predicted probability of the test data, and then the entropy of the predicted probability is used as the OOD detection score. Both SPBN and temperature scaling are applied here only in the feature learner and classifier.

#### 3.3.1. Self perturbation batch normalization

Our proposed SPBN normalizes the input data in the inference process similar to the training process of standard BN. Batch normalization as a regularization technique with state-of-the-art network structure is widely used (He et al., 2016; Huang et al., 2017). It can accelerate the training process of deep neural networks by rescaling the mean and variance of activation values in the BN layer (Ioffe and Szegedy, 2015). The rescaling process consists of two steps: normalization, and scaling. The standard BN is normalized in the training phase using the statistical values of the current input data. Still, instead of using the existing data, the fixed statistical values estimated from the training data are used in the inference phase. The standard BN assumes that both the test data and the training data belong to the ID distribution, while our task encounters OOD data that do not belong to the ID distribution. Our approach exploits this by no longer using fixed statistical values $\hat{\mu },\hat{\sigma }$ to normalize the input data during inference, but rather using the statistical values μ_*B*_, σ_*B*_ of the current data. The differences between the statistical values of the current data are used to perturb the predictions.

Given the *d*-dimensional input data $x_{i}=\left(x_{i}^{\left(1\right)},x_{i}^{\left(2\right)},...,x_{i}^{\left(d\right)}\right)$, normalization is applied to *x*_*i*_ using the current batch of data $X_{B}={\left\{x_{i}\right\}}_{i=1}^{n}$ by using (5),


(5) $$\begin{array}{l} {\hat{x}}_{i}=\frac{x_{i}-\mu_{B}}{\sqrt{\sigma_{B}^{2}+\epsilon }} \end{array}$$

where $\mu_{B}=\frac{1}{n}\overset{n}{\underset{i=1}{\sum }}x_{i},\sigma_{B}^{2}=\frac{1}{n}\overset{n}{\underset{i=1}{\sum }}{\left(x_{i}-\mu_{B}\right)}^{2}$. The second step scales the normalized activation values using the parameters in the BN layer, which are learned during the training process and are not changed during the inference process. Using the trained BN layer parameters γ = [γ^(1)^, γ^(2)^, ..., γ^(*d*)^] and β = [β^(1)^, β^(2)^, ..., β^(*d*)^] are scaled and biased for $\tilde{x}$ to obtain the corrected value ${\tilde{x}}_{i}$, which is calculated as (6)


(6) $$\begin{array}{l} {\tilde{x}}_{i}^{\left(k\right)}=\gamma^{\left(k\right)}{\hat{x}}_{i}^{\left(k\right)}+\beta^{\left(k\right)},k=\left\{1,2,...,d\right\} \end{array}$$

#### 3.3.2. Temperature scaling

Temperature scaling changes the predicted probability distribution of the model through the temperature parameter *T* (Hinton et al., 2015). It not only corrects the predicted probability distribution of the model output (Guo et al., 2017), but also alleviates the problem of overconfidence of the model (Liang et al., 2018). Logits *z*_*i, h*_ of sample *x*_*i*_ with respect to category *h* and predicted probability ŷ_*i, h*_ are calculated as shown in Equation (7)


(7) $$\begin{array}{l} {ŷ}_{i,h}=\frac{\exp\left(z_{i,h}/T\right)}{\sum_{h=1}^{C}\exp\left(z_{i,h}/T\right)} \end{array}$$

When *T*≥1, the probability vector becomes smooth. Further studies (Liang et al., 2018) found that larger *T* is used (e.g., *T*≥1,000), higher order differences between logits components are masked out, which affects the OOD detection performance of the model. In this paper, we use temperature scaling to further amplify the difference between ID and OOD data.

## 4. Experiments

In this section, we validate the effectiveness of the MPSE method on semantic enhancement and OOD detection performance using four experiments. Section 4.1 visualizes the OOD generalization capability of multiple “semantic perspectives” using a toy dataset; Section 4.2 gives the metrics and comparison methods for the OOD detection task; Section 4.3 illustrates the effectiveness of the MPSE approach in semantic enhancement using the MNIST dataset; Section 4.4 conducts experiments on the OOD benchmark dataset to verify the performance of the model to detect OOD and SMOOD data, and ablation experiments; Section 4.5 verifies the performance of MPSE on larger size images on the Oxford flowers102 dataset.

Our approach uses the corresponding basic network for training and learning in different experiments based on different datasets, but all use the same fusion network. The fusion network uses a hidden layer of perceptron as the backbone network. The hidden layer dimension is set to 1,000, and 100 epochs are trained. The optimizer uses the SGD algorithm, and the initial learning rate is 0.1, which is reduced to 0.01 and 0.001 after epochs*0.5 and epochs*0.75, respectively, and the Dropout is set to 0. Our method sets the number of semantic perspectives to five in training and the coefficient of reconstruction loss to 0.01. Since our method uses cos similarity to calculate logits, the temperature coefficient is set to 25 to avoid numerical problems.

### 4.1. Toy dataset experiment

**Figure 2:**
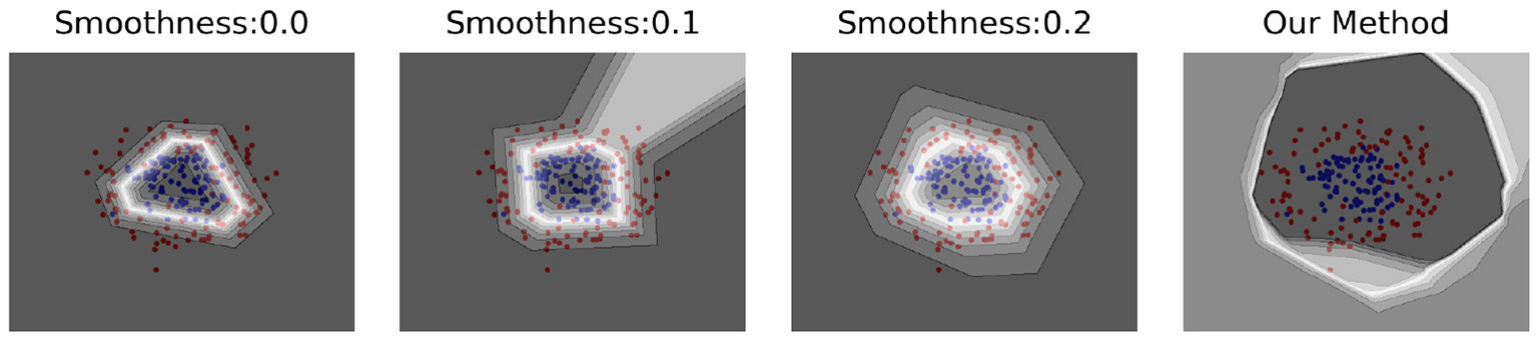
The effect of this method with the toy dataset. This toy dataset has two dimensions, represented by red and blue dots in a ring-like distribution with inconsistent diameters. The two rings have a diameter ratio of 0.4 and are generated using a Gaussian noise of 0.2. A gradient color from black to white is used to represent the maximum value predicted by the model for that coordinate point, with darker colors representing larger values. The first three subplots are models obtained under different “semantic perspectives,” which are trained using smoothing labels with smoothing coefficients of 0.0, 0.1, and 0.2, respectively. The rightmost subplot shows the model learned using our method without SPBN for the three “semantic perspectives.” Obviously, our method more reasonably discriminates the region outside the two circles as a low confidence region.

Our method was validated on a ring-shaped toy dataset, as shown in Figure 2. We can observe that the models based on a single “semantic view” predict the inter-class boundaries as low-confidence regions, i.e., all spaces outside the outer ring data are considered as high-confidence ID data. Using our method, we can clearly observe that the confidence level of the inner data of the two loops is significantly higher than that of the outer region, indicating that our method can better characterize the overall boundaries of ID data and make more accurate predictions for OOD samples after incorporating multiple “semantic perspectives.”

### 4.2. Metrics and comparison methods

#### 4.2.1. Performance metrics

The two most commonly used metrics in OOD detection tasks are AUROC and TNR@TPR95, which represent a better model when they are higher. To calculate the AUROC of the model, the first step is to consider the identification of ID and OOD samples as a binary classification problem, and then calculate the True Positive Rate (TPR) and False Positive Rate (FPR) under each threshold, respectively, and lastly plot the curve and calculate the area under the curve based on the values of TPR and FPR. TNR@TPR95 calculates the value of 1-FPR when TPR = 95% for the ID dataset, which indicates the identification rate of the OOD dataset when the identification rate of the ID dataset reaches 95%.

#### 4.2.2. Comparison methods

The comparison methods include Baseline (Hendrycks and Gimpel, 2017), ODIN (Liang et al., 2018), and Deconf-c in GODIN (Hsu et al., 2020), where ODIN and Deconf-c use the methods in GODIN to perturb the input test data, noted as ODIN* and Deconf-c*. Our methods include Multi-Perspective Semantic Enhancement (MPSE), MPSE-attn, and MPSE-both, MPSE-attn adds the attention mechanism and MPSE-both incorporates reconstruction regular term with the attention mechanism.

### 4.3. Semantic enhancement experiments

To verify the effectiveness of our method in extracting and maintaining semantic information, this experiment divides OOD data into two types: OOD data with the same semantics as ID data, and the rest as the other type. The first type of data has different distribution from the ID data, but the semantics is the same, and they belong to the same class, which we refer to as the same class OOD (SCOOD) data. In contrast, another type we simply call the different class of OOD (DCOOD) data.

In this subsection, we first learn to obtain the model on ID data (training set), and then test the performance of the model using ID data (test set), SCOOD data, and DCOOD data. The test consists of three parts: (1) the ability of the model to distinguish ID data from OOD data (both SCOOD and DCOOD); (2) the ability of the model to distinguish SCOOD from DCOOD; and (3) the classification performance of the model on ID and SCOOD.

#### 4.3.1. Datasets and training details

We use MNIST dataset (LeCun et al., 1998) as ID data. there are 10 categories in MNIST, representing 0–9 10 handwritten digits; there are 50,000 samples in training set, 5,000 samples in each class; there are 10,000 samples in test set, 1,000 samples in each class. Each sample is a 28*28 image of a single channel. We use USPS dataset (Hull, 1994) as SCOOD data. USPS is also a handwritten digit set with 0–9 classes, and the whole dataset has 9,298 samples. Each sample is a single channel image of 16*16, in using resize the image to 28*28 size. The rest DCOOD data uses FashionMNIST (Xiao et al., 2017), omniglot (Lake et al., 2015), cifar10-bw (Hendrycks and Gimpel, 2017), and two noise datasets, Gaussian noise and uniform noise, respectively. Each dataset is 10,000 samples, with the same size and channels as MNIST.

In this experiment, we use Lenet as the backbone network. Lenet was trained for 20 epochs using a batch size of 128 and a weight decay of 0.0005 during training.

#### 4.3.2. Experiment results and analysis

The OOD (both SCOOD and DCOOD) detection performance results of the model are shown in Table 1. It can be observed that (1) in the detection of SCOOD data, all methods are able to discriminate better, indicating that USPS and MNIST do belong to different distributions; (2) in all OOD data, our method significantly outperforms the comparison method, showing excellent performance in the more difficult tnr@tpr95 metric and more robust in noise detection in particular.

**Table 1:** Performance of the different methods in OOD (both SCOOD and DCOOD) scenarios, with the best results shown in bold.

**ID**	**OOD**	**AUROC**	**TNR@TPR95**
		Baseline/ODIN*/Deconf-C*/ MPSE/ MPSE-attn/ MPSE-both	
MNIST	USPS	89.2/92.9/95.0/99.1/**99.3**/98.8	62.8/66.0/72.8/98.5/**99.6**/96.6
	Fashion-MNIST	94.8/96.8/97.4/**99.9/99.9/99.9**	76.9/83.4/88.8/**100./100./99.9**
	Omniglot	91.3/93.7/93.5/**100./100./100**.	51.2/61.0/71.8/**100./100./100**.
	Cifar10-bw	92.2/93.1/99.6/**100./100./100**.	70.6/70.9/98.9/**100./100./100**.
	Uniform	78.0/78.6/75.5/**100./100./100**.	19.0/1.10/27.9/**100./100./100**.
	Gaussian	28.5/43.4/66.9/**100./100./100**.	0.00/0.00/0.30/**100./100./100**.

All results are averaged after 3 runs using the model.

Table 2 shows the results of the model learned in the ID data to distinguish between SCOOD and DCOOD types of data. The results show that our approach is still able to discriminate well-between the different types of OOD data, except for the comparison approach, which fails almost completely. It shows that learning under a single “semantic perspective” is corrupted by a large amount of non-semantic information; while our method learns under multiple “semantic perspectives” and obtains more comprehensive and effective semantic information. The results in Table 3 further confirm the above conclusions. Although all methods were able to obtain excellent accuracy in the test set of ID data, only our method maintained a high accuracy in the SCOOD dataset with the same class semantics.

**Table 2:** The model was trained using only the ID dataset (MNIST) to discriminate the performance of SCOOD against DCOOD data, and the best results are shown in bold.

**SCOOD**	**DCOOD**	**AUROC**	**TNR@TPR95**
		Baseline/ODIN*/Deconf-C*/ MPSE/ MPSE-attn/ MPSE-both	
USPS	Fashion-MNIST	59.6/62.3/62.4/84.6/84.2/**86.2**	6.20/4.50/8.60/38.4/37.0/**40.8**
	Omniglot	43.2/43.2/46.4/97.6/**98.1**/97.9	0.50/0.20/6.20/85.2/**88.2**/87.1
	Cifar10-bw	56.7/59.3/84.0/95.0/**95.2**/94.9	4.10/4.10/29.9/72.7/**74.0**/72.3
	Uniform	22.4/14.8/18.9/99.4/**99.6/99.6**	0.00/0.00/0.00/97.5/98.5/**98.7**
	Gaussian	4.30/4.50/3.40/99.7/**99.9/99.9**	0.00/0.00/0.00/99.2/**99.7/99.7**

All results are averaged after 3 runs using the model.

**Table 3:** Classification accuracy of the model after training using the ID dataset (MNIST) on ID and SCOOD data, with the best results shown in bold.

**TestData**	**Accuracy**					
	**Baseline**	ODIN*	Deconf-C*	**MPSE**	**MPSE-attn**	**MPSE-both**
MNIST (ID)	99.2	99.2	99.3	**99.5**	**99.5**	**99.5**
USPS (SCOOD)	61.5	61.5	48.3	89.1	88.2	**89.4**

All results are averaged after 3 runs using the model.

### 4.4. OOD and SMOOD data detection experiments

In Section 4.3, OOD data are classified into SCOOD and DCOOD types according to semantics, This section does not consider the semantics of OOD data and ID data, but studies the similarity of the distribution between them. We calculate the MMD distances between different datasets according to the method provided in the literature (Sutherland et al., 2016; Liang et al., 2018), and consider that OOD data with particularly small MMD distances have similar statistical features to ID data, and refer to such OOD data as SMOOD data. Given two image sets *V* = *v*_1_, *v*_2_, ..., *v*_*m*_ and *W* = *w*_1_, *w*_2_, ..., *w*_*m*_, the MMD distance is calculated as shown in Equation (8)


(8) $$\begin{array}{l} {\hat{MMD}}^{2}(V,W)=\frac{1}{\left(\overset{m}{2}\right)}\sum_{i\neq j}k(v_{i},v_{j})+\frac{1}{\left(\overset{m}{2}\right)}\sum_{i\neq j}k(w_{i},w_{j})\\ \;-\frac{2}{\left(\overset{m}{2}\right)}\sum_{i\neq j}k(v_{i},w_{j}) \end{array}$$

where *k*(·, ·) is the Gaussian RBF kernel function, i.e., $k\left(x,x^{\prime }\right)=\exp\left(-\frac{∥x-x^{\prime }{∥}_{2}^{2}}{2\sigma^{2}}\right)$. 2σ^2^ uses the median of the set *V*∪*W* of all Eulerian distances. In the next experiments, we first study the performance of the model on the OOD benchmark dataset, and then on the SMOOD data.

#### 4.4.1. Datasets and training details

In this experiment, following the setup of Hsu et al. (2020), Cifar10/Cifar100 was used for the ID data, and the OOD data consisted of eight datasets, namely Imagenet(crop), Imagenet(resize), LSUN(crop), LSUN(resize), iSUN, Gussian Noise, Uniform Noise, and SVHN datasets. Cifar10/100 has 10/100 classes, 5,000/500 samples per class in the training set, and 1,000/100 samples per class in the test set. Ten thousand samples in the OOD data, except for iSUN, which has only 8,925 samples. All images are 32*32*3 in size. Our method was applied to two types of networks, ResNet and DenseNet. ResNet, with a depth of 34, was trained for 200 epochs using a batch size of 128 and a weight decay of 0.0005 during training decay. The depth of DenseNet is set to 100 layers, the growth rate is 12, the training process batch size is 64, the weight decay is set to 0.0001, and 300 epochs are trained.

#### 4.4.2. OOD benchmark results and analysis

Results for the comparison methods Baseline, ODIN*, and Deconf-c* are from Hsu et al. (2020), the results of our method use the mean value of the model after 3 runs. Table 4, which shows the average values on the eight OOD datasets. The data in Table 4 show that our method significantly outperforms the other methods, substantially outperforming the compared methods on both the cifar10 and cifar100 datasets. It should be noted that Deconf-c* uses sample perturbation for preprocessing, and our method also outperforms the Deconf-c* method in terms of the average value of OOD data detection metrics across the board without preprocessing. In addition, our algorithm significantly outperforms the comparison method in terms of classification accuracy of ID data because it learns the semantic information of ID data more effectively and comprehensively. It can also be observed from Table 4 that the best ID accuracy is obtained using only nonlinear transformations in the feature learner, but the OOD generalization performance on the Cifar10 dataset is not as good as the model with the addition of the attention mechanism and reconstructed regular terms. It is indicated that it helps to maintain the valid semantic information learned by the model with rich samples and avoid overfitting of the model to ID non-semantic information.

**Table 4:** Performance of different methods on OOD benchmark dataset with semantic extraction using ResNet/DenseNet as the bone network, best results are shown in bold.

**ID**	**NET**	**AUROC**	**TNR@TPR95**	**ID Accuracy**
		Baseline/ODIN*/Deconf-C*/ MPSE/ MPSE-attn/ MPSE-both		
Cifar10	ResNet	89.3/83.6/97.8/98.7/98.9/**99.0**	49.9/60.0/89.9/91.6/91.9/**94.2**	95.2/95.2/95.1/**95.8**/95.6/95.2
	DenseNet	92.6/92.7/99.2/98.9/99.0/**99.3**	50.9/64.8/96.2/92.2/92.4/**96.5**	95.2/95.2/95.0/**95.9**/95.8/95.7
Cifar100	ResNet	72.4/86.7/96.2/**98.7**/98.4/96.7	15.6/46.0/77.8/**93.2**/90.5/82.9	78.5/78.5/75.8/**79.1**/77.6/77.3
	DenseNet	72.5/86.9/98.0/98.6/**98.7**/89.2	17.3/53.2/89.9/**95.1**/93.2/59.8	77.0/77.0/75.9/**80.7**/78.7/73.0

Results for the AUROC and TNR@TPR95 metrics were averaged from the eight OOD datasets, and accuracy was obtained from the test set of ID data. All results are the mean values after 3 runs using the model.

#### 4.4.3. SMOOD results and analysis

In this experiment, we focus on verifying the performance of our method for detecting SMOOD data. Table 5 shows the MMD distances between the different data sets, and the MMD distances are calculated using Equation (8). Obviously, the MMD distance between the two datasets Cifar10 and Cifar100 is much smaller than the other datasets. Here they are used mutually as SMOOD data to test the performance of the model. SMOOD data has similar semantics to ID data, and this similar semantic information can interfere with the model's detection of OOD data, thus the detection of near OOD data is more challenging in the OOD detection task. The results are shown in Table 6, all results are the mean values after 3 runs using the model. We found that the detection performance of ODIN*, Deconf-c* methods did not improve compared to baseline on SMOOD data, and even decreased, indicating that the features learned by the model under a single “perspective” have limited semantic information, which is further corrupted by the sample perturbation and feature statistics methods. Our method greatly outperforms the comparison method on both ResNet and DenseNet, except for the version that adds refactored regular terms. The reconstruction regular term has a positive impact on Cifar10, showing the best detection performance, however, a negative impact is observed on Cifar100. This is due to the limited accuracy of the Cifar100's feature extractor itself, the extracted features do not accurately represent the semantic information of the ID data, and the reconstruction regular term hinders the feature learner from further optimizing to learn more accurate semantic information. The overall results show that our method learns from multiple “semantic perspectives” and the features extracted by the model contain richer discriminative information, which helps the model to detect SMOOD data.

**Table 5:** The maximum mean distance between ID data and different OOD data, the smallest distance is shown using bold and “–” represents a possible case of semantic overlap and is therefore not computed.

	**Img(c)**	**Img(r)**	**LSUN(c)**	**LSUN(r)**	**iSUN**	**Gaussian**	**Uniform**	**SVHN**	**Cifar100**	**Cifar10**
Cifar10	14.0	0.65	14.9	1.00	0.63	6.72	1.98	2.54	**0.14**	–
Cifar100	13.6	0.68	14.4	0.88	0.60	5.90	1.89	2.88	–	**0.15**

**Table 6:** Performance of different methods on SMOOD data with semantic extraction using ResNet as the bone network, best results are shown in bold.

**ID**	**OOD**	**NET**	**AUROC**	**TNR@TPR95**
			Baseline/ODIN*/Deconf-C*/ MPSE/ MPSE-attn/ MPSE-both	
Cifar10	Cifar100	ResNet	87.5/71.9/87.3/90.2/88.5/**92.9**	37.0/25.6/49.2/**54.9**/51.5/52.8
		DenseNet	89.1/74.7/85.3/91.3/92.3/**93.8**	39.1/30.3/39.4/55.3/54.3/**57.4**
Cifar100	Cifar10	ResNet	75.7/72.3/73.5/**92.9**/92.7/84.9	16.3/14.2/16.3/51.0/**51.1**/23.9
		DenseNet	77.2/64.2/73.2/88.7/**91.2**/66.3	18.6/9.41/17.4/**52.7**/47.4/17.2

All results are the mean values after 3 runs using the model.

#### 4.4.4. Parameter selection

This experiment verifies the impact of different numbers of “semantic perspectives” and temperature scaling coefficient, using the more difficult to detect SMOOD data for ID and OOD data, corresponding to Cifar100 and Cifar10, and ResNet as the skeleton network for feature extraction.

**Figure 3:**
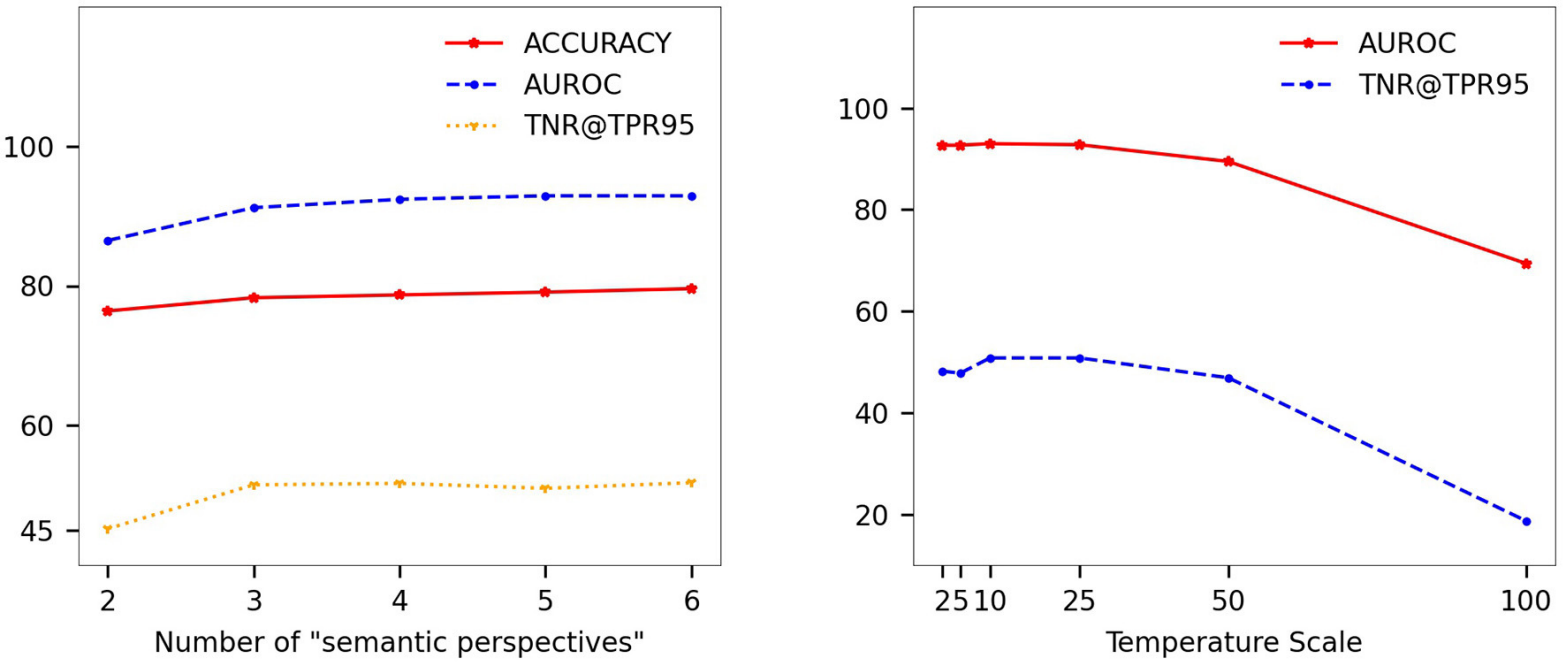
Regarding the effect of three parameters on the model performance: the number of “semantic views,” and the temperature scaling coefficient. The ID and OOD data used in this experiment correspond to Cifar100 and Cifar10, and the skeleton network for feature extraction is ResNet. **(Left)** Selection of the number of “semantic perspectives.” **(Right)** Effect of different temperature scaling coefficients on the model.

As you can see from Figure 3 on the left, the number of “semantic perspectives” increases, the accuracy of ID data, the AUROC metric of OOD data and the TNR@TPR95 metric improve, but the improvement becomes slower as the number increases. We believe that the number of perspectives set to 5 is sufficient to demonstrate the effectiveness of the method.

In the Figure 3 on the right, a slight temperature scaling has some improvement effect, but a slightly larger scaling factor leads to a decrease in performance. It is the number of categories that are too large (100) that causes numerical problems when calculating entropy.

#### 4.4.5. Ablation study

**Figure 4:**
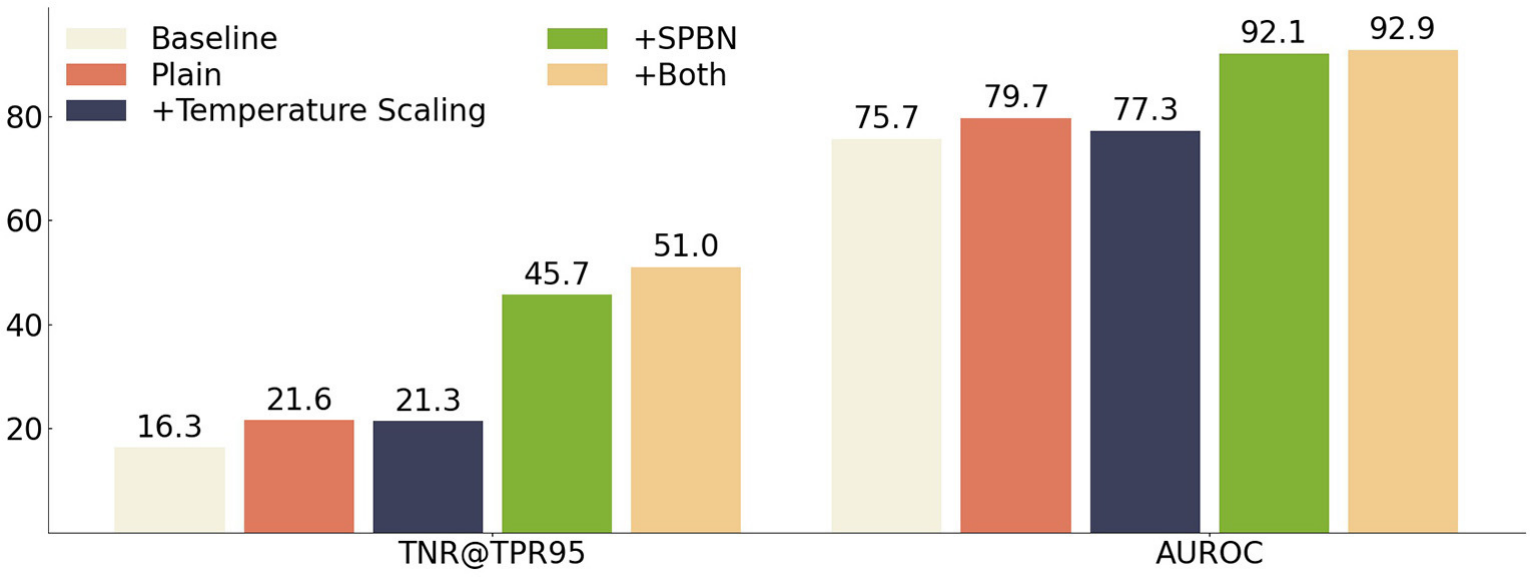
The effects of SPBN, temperature scaling SMOOD data are demonstrated, using both TNR@TPR95 and AUROC metrics. The feature extractor is trained using ResNet as the bone network, using the method proposed in this paper. The ID, OOD datasets for training and testing the model use Cifar100 and Cifar10, respectively.

Our method applies both SPBN and temperature scaling techniques in the inference. In this experiment ResNet is used as the feature extraction network, and Cifar100 and Cifar10 are used for ID and OOD data, respectively, to study their effects on the model. Figure 4 shows the impact caused by different inference tricks. Our approach outperforms the baseline approach in all cases, using the full inference trick to achieve the best performance on the test set. The ablation of SPBN has a significant impact on our approach, suggesting that multiple “semantic perspective” feature statistics play an important role in the learning process of fused feature semantics. The temperature scaling has a negative impact on the SMOOD dataset when used alone, because the model uses the wrong statistics without applying SPBN, and still improves the model performance slightly when SPBN is applied.

### 4.5. Large size dataset experiment

Unlike the previous use of a small size (32*32) input image, this section verifies the performance of the proposed method on a larger size (224*224) input image. In the experiments, the MMD distances of ID data and OOD data are first given, and then reported for the two metrics AUROC and TNR@TPR95.

#### 4.5.1. Datasets and training details

In this experiment ID data and SMOOD data were used in the Oxford Flowers102 dataset (Nilsback and Zisserman, 2008). This dataset has 102 categories with the number of each category ranging from 40 to 258, for a total of 8,189 samples. The short edge of each sample is 500 pixels. ID data used the first 90 classes, noted as Flowers90, with a total of 7,254 samples, of which 1,000 samples were randomly divided as the test set and the rest as the training set; SMOOD data used the last 12 classes, Flowers12, with a total of 935 samples. The remaining OOD data are also available in four datasets, namely the CUB200-2011 Bird dataset (Wah et al., 2011), the Stanford Car dataset (Krause et al., 2013), Gaussian noise, and uniform noise. Each is 1,000 samples. Resnet-18 was selected as the backbone network for the experiment, was trained for 200 epochs using a batch size of 256 and a weight decay of 0.0005 during training decay. For model inference, a batch size of 200 is used.

#### 4.5.2. Experiment results and analysis

First, the results in Table 7 show that the MMD distance for flowers12 is much lower than the rest of the OOD data set, indicating similar statistical properties to the ID data. Second, the ODIN method obtains worse performance than the baseline method in the absence of the OOD data tuning perturbation magnitude parameter; while the GODIN method enhances the performance of detecting OOD data, its performance decreases instead when encountering SMOOD type data. Our approach is able to further improve performance beyond the baseline approach even in the face of SMOOD data. Finally, it is worth mentioning that our method exhibits excellent performance when detecting noise.

**Table 7:** Performance of different methods on large size image data (Flowers90).

**OOD**	**MMD**	**AUROC**	**TNR@TPR95**
		Baseline/ODIN*/Deconf-C*/MPSE/MPSE-attn/MPSE-both	
CUB200-2011	5.85	83.7/80.9/**94.4**/86.6/81.9/87.2	31.1/42.8/**70.4**/38.2/36.0/43.8
Stanford Car	6.69	84.2/82.8/91.1/95.2/94.4/**96.5**	28.4/29.6/48.3/74.7/74.5/**80.3**
Gaussian	9.30	49.0/3.80/58.9/99.9/**100**./99.9	0.00/0.00/0.00/99.9/**100**./99.9
Uniform	7.09	77.3/7.70/67.7/**100./100**./99.9	0.00/0.00/0.00/**100./100**./99.7
Flowers12	0.71	78.8/66.9/73.0/80.9/79.3/**83.5**	17.5/8.60/9.50/22.1/25.0/**27.3**

Resnet18 is used as the backbone network. Best results are shown in bold, all results are the mean values after 3 runs using the model.

## 5. Conclusion

Our proposed multiple “semantic perspectives” approach is simple to train, as it is model-irrelevant in the feature extraction phase, and only requires a set of labels with the correct semantics when extracting different “semantic perspectives”; and in the feature fusion phase, it only utilizes In the feature fusion phase, a simple perceptron network structure is utilized. Our proposed SPBN perturbation method does not require any preprocessing of the input images for model inference, and only utilizes the different normalized mean values of the BN layers. However, this also limits the scope of application of the SPBN method to network structures containing BN layers.

Our proposed “multi-perspective” semantic enhancement and SPBN strategy can learn more comprehensive and accurate semantic information from ID data, which can effectively resist the interference of SMOOD data and improve the performance of the model in detecting OOD data. Our method works well not only for small size images but also for larger ones.

## Data availability statement

The original contributions presented in the study are included in the article/supplementary material, further inquiries can be directed to the corresponding author/s.

## Author contributions

YY: propose ideas for papers, guide the writing of papers, and propose grants for papers. WJ: complete experiments, compile and analyze experimental results, and finish writing of papers. Both authors contributed to the article and approved the submitted version.

## Funding

This work was supported by National Natural Science Foundation of China (NSFC) under grant 61873067 and the University-Industry Cooperation Project of Fujian Provincial Department of Science and Technology under grant 2020H6101.

## Conflict of interest

The authors declare that the research was conducted in the absence of any commercial or financial relationships that could be construed as a potential conflict of interest.

## Publisher's note

All claims expressed in this article are solely those of the authors and do not necessarily represent those of their affiliated organizations, or those of the publisher, the editors and the reviewers. Any product that may be evaluated in this article, or claim that may be made by its manufacturer, is not guaranteed or endorsed by the publisher.
